# Adrenal Hemorrhage in a Cortisol-Secreting Adenoma Caused by Antiphospholipid Syndrome Revealed by Clinical and Pathological Investigations: A Case Report

**DOI:** 10.3389/fendo.2021.769450

**Published:** 2022-02-03

**Authors:** Kentaro Ochi, Ichiro Abe, Yuto Yamazaki, Mai Nagata, Yuki Senda, Kaori Takeshita, Midori Koga, Yuka Yamao, Toru Shigeoka, Tadachika Kudo, Yuichiro Fukuhara, Shigero Miyajima, Hiroshi Taira, Shoji Haraoka, Tatsu Ishii, Yuichi Takashi, Alfred K. Lam, Hironobu Sasano, Kunihisa Kobayashi

**Affiliations:** ^1^ Department of Endocrinology and Diabetes Mellitus, Fukuoka University Chikushi Hospital, Chikushino, Japan; ^2^ School of Medicine and Dentistry, Griffith University, Gold Coast, QLD, Australia; ^3^ Department of Pathology, Tohoku University Graduate School of Medicine, Sendai, Japan; ^4^ Department of Urology, Fukuoka University Chikushi Hospital, Chikushino, Japan; ^5^ Department of Pathology, Fukuoka University Chikushi Hospital, Chikushino, Japan

**Keywords:** adrenal hemorrhage, cortisol-secreting adenoma, antiphospholipid syndrome, thrombosis, thrombophilia

## Abstract

Due to its rarity, adrenal hemorrhage is difficult to diagnose, and its precise etiology has remained unknown. One of the pivotal mechanisms of adrenal hemorrhage is the thrombosis of the adrenal vein, which could be due to thrombophilia. However, detailed pathological evaluation of resected adrenal glands is usually required for definitive diagnosis. Here, we report a case of a cortisol-secreting adenoma with concomitant foci of hemorrhage due to antiphospholipid syndrome diagnosed both clinically and pathologically. In addition, the tumor in this case was pathologically diagnosed as cortisol-secreting adenoma, although the patient did not necessarily fulfill the clinical diagnostic criteria of full-blown Cushing or sub-clinical Cushing syndrome during the clinical course, which also did highlight the importance of detailed histopathological investigations of resected adrenocortical lesions.

## Introduction

Adrenal hemorrhage is rare and caused by various etiologies (1, 2). Systemic studies demonstrated that abdominal trauma, infections, surgery, angiography, and adrenal venous thrombosis could cause adrenal hemorrhage (1–6). As such, it is difficult to diagnose and determine its etiology solely through clinical investigations. In addition, only a few cases have been reported on their pathological findings. In addition, histopathology of adrenal hemorrhage in adrenocortical adenoma has not been described in the literature. Herein, we report the first case of adrenal hemorrhage in a cortisol-secreting adenoma due to thrombophilia by antiphospholipid syndrome.

## Case Presentation

### Clinical Summary

**Figure 1:**
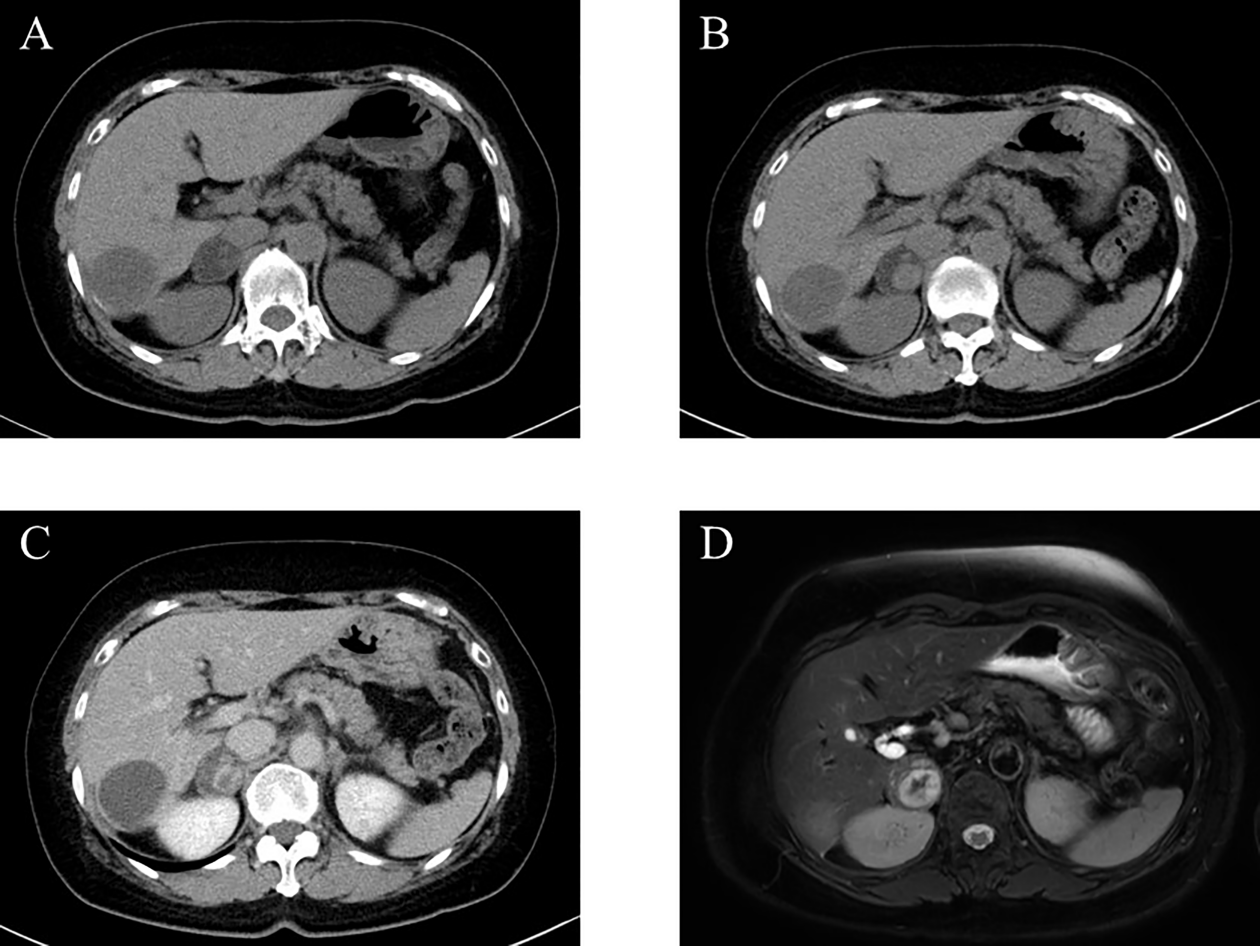
Imaging analysis. **(A)** Non-enhanced computed tomography (CT) at the first admission. **(B, C)** Non-enhanced **(B)** and enhanced **(C)** CT 3 years after the first admission. **(D)** T2-weighted magnetic resonance imaging (MRI) at the second admission.

A 67-year-old woman was incidentally diagnosed with a nodular lesion in her right adrenal gland (25 × 29 mm) by non-enhanced abdominal computed tomography (CT) (**Figure 1A**). She was referred to our hospital and was admitted for further endocrinological examination. Her body mass index was 26.0 kg/m^2^, and she did not have any clinical findings, suggestive of Cushing’s syndrome. Her blood pressure was within normal limits (109/51 mmHg). In addition, she did not have hyperlipidemia [low-density lipoprotein (LDL)-cholesterol, 134 mg/dL; high-density lipoprotein (HDL)-cholesterol, 58 mg/dL; triglyceride 79 mg/dL). Her HbA1c was 5.5%. Results of those laboratory findings were almost within the normal range, but her potassium level was slightly low (3.7 mmol/L). She had never received any anticoagulant therapy and medication for hypertension, hyperlipidemia, and diabetes mellitus in her past history. In the endocrine evaluation, her morning adrenocorticotropic hormone (ACTH) level was relatively low (5.0 pg/mL), while her cortisol level was within the normal range (12.60 µg/dL). Results of 1-mg dexamethasone suppression test demonstrated serum cortisol suppression (0.89 µg/dL), which was lower than 1.8 µg/dL. Her nocturnal serum cortisol levels (taken at 21 pm after 30 min resting) were not necessarily high (2.68 µg/dL), which was lower than 5.0 µg/dL. Dehydroepiandrosterone sulfate level was within normal limits (28.0 µg/dL; normal range, 12–133 µg/dL). These results did not necessarily meet the clinical diagnostic criteria of full-brown Cushing’ syndrome (CS) and subclinical Cushing’s syndrome (SCS) (7). Her plasma renin activity was low (0.2 ng/mL/hr), while her plasma aldosterone concentration was not necessarily high (80.8 pg/mL; normal range, 4.0–82.1 pg/mL). The ratio of plasma aldosterone concentration/plasma renin activity (ARR) was 404. Results of the upright furosemide-loading test met the diagnostic criteria of primary aldosteronism, while those of the captopril-challenge test and the saline-loading test were within the normal range (**Table 1**). These results above were not conclusive of the clinical diagnosis of primary aldosteronism (7, 8). The plasma/urinary catecholamine and urinary metanephrines values were within the normal range. However, the simultaneous presence of autonomous cortisol secretion and primary aldosteronism could not completely be ruled out in this case. Therefore, the patient was carefully surveyed following her first admission. Three years after her admission, abdominal non-enhanced and enhanced CT demonstrated that the right adrenal tumor had enlarged (35 × 40 mm) and became heterogeneous (**Figures 1B, C**). Therefore, she was readmitted for further endocrinological examination.

**Table 1:** Summary of endocrine disorders of the patient.

		1st admission	Preoperation	3 months after operation	The diagnostic criterion of CS/SCS
presence or absence of the physical symptom of Cushing’s syndrome		Absence	Absence	Absence	CS: presence/SCS: absence
Morning plasma ACTH level (pg/mL)		5.0	8.7	25.8	CS: <5.0 pg/mL/SCS: <10.0 pg/mL
Morning serum cortisol level (µg/dL)		12.60	9.01	5.62	CS: normal or high (> 8 µg/dl)/SCS: normal (8–18 µg/dL)
Nocturnal serum cortisol level (µg/dL)		2.68	3.84	N.A.	CS: <7.5 µg/dL/SCS: < 5.0 µg/dL
Serum cortisol level on 1-mg dexamethasone suppression test (µg/dL)		0.89	1.38	0.69	CS: <5.0 pg/mL/SCS: <1.8 pg/mL
Dehydroepiandrosterone sulphate level (µg/dL)		28	15	18	CS and SCS: < 12 µg/dL (considering patient’s sex and age)
		**1st admission**	**Preoperation**	**3 months after operation**	**The diagnostic criterion of primary aldosteronism**
Baseline ARR		404	381	369	ARR > 200
Captopril-challenge tests	ARR (60 min)	56	189	199	ARR (60 or 90 min) > 200
	ARR (90 min)	41	248	182	
upright furosemide-loading tests	plasma renin activity (120 min) (ng/mL/hr)	0.9	0.9	1.2	Plasma renin activity (120 min) < 2.0 ng/mL/hr
Saline-loading test	Plasma aldosterone (240 min) (pg/mL)	11.1	<10.0	21.4	Plasma aldosterone (240 min) > 60 pg/mL

CS, Cushing’s syndrome; SCS, subclinical Cushing’s syndrome; N.A, not assessed; ARR, the ratio of plasma aldosterone concentration/plasma renin activity.

Her body mass index was 26.6 kg/m^2^, and she did not have any clinical findings suggestive of Cushing’s syndrome, same as the first admission. She remained normotensive (106/62 mmHg) and without hyperlipidemia and diabetes mellitus. Her potassium level was 3.6 mmol/L. In the endocrine evaluation, her morning ACTH level was still low (8.7 pg/mL), but the cortisol level was within the normal range (9.01 µg/dL). Results of 1-mg dexamethasone suppression test at this admission demonstrated the serum cortisol value higher than that in her first admission (1.38 µg/dL). Besides, her nocturnal serum cortisol level (taken at 21 pm after 30 min resting) was slightly high (3.84 µg/dL). Meanwhile, dehydroepiandrosterone sulfate level was relatively low (15.0 µg/dL). In addition, ^131^I-adosterol adrenal scintigraphy showed high uptake of adosterol in the tumor on the right adrenal gland and suppressed uptake in the left adrenal gland. These results indicated that the tumor could be a cortisol-secreting adrenocortical adenoma despite having insufficient diagnostic criteria of SCS. Her plasma renin activity was low (0.2 ng/mL/hr), and her plasma aldosterone concentration was not high (76.2 pg/mL), with an ARR of 381. Results of the captopril-challenge tests and upright furosemide-loading tests satisfied the diagnostic criteria of primary aldosteronism. Results of the saline-loading test were within normal range, same as the first admission (**Table 1**). Overall, the patient could have primary aldosteronism.

Abdominal magnetic resonance imaging (MRI) was performed for the patient and showed a high-intensity region inside the tumor, suspected to be adrenal hemorrhage, despite having no abdominal pain (**Figure 1D**). In addition, she had a history of repeated miscarriage and was positive for anticardiolipin antibody (21.0 U/mL). Based on those findings above, antiphospholipid syndrome was clinically diagnosed.

Considering the tumor enlargement and the clinical necessity of anticoagulant therapy, a laparoscopic resection was performed on the right adrenal tumor. A laparoscopic resection was on the right adrenal tumor. After the operation, she was placed in hydrocortisone replacement therapy. However, her cortisol level remained low (1.14 µg/dL) 14 days after the operation. Three months after the operation, her ACTH and cortisol levels were 25.8 pg/mL and 5.62 µg/dL, respectively. In addition, results of 1-mg dexamethasone suppression test adequately demonstrated cortisol suppression (0.69 µg/dL), and dehydroepiandrosterone sulfate level increased (18.0 µg/dL) (**Table 1**). Therefore, the replacement of hydrocortisone was tentatively terminated at that time. Six months after operation, her ACTH and cortisol levels became normal (33.5 pg/mL and 10.1 µg/dL, respectively). However, there were no changes of ARR and results of the upright furosemide-loading test before and 3 months after operation. The captopril challenge test demonstrated that there were only slight but not significant differences of ARR between before and 3 months after operation (**Table 1**). Therefore, the possibility of primary aldosteronism of the right adrenocortical tumor could be excluded, and the culprit lesion of primary aldosteronism could exist in the left adrenal gland. Regarding the treatment of her antiphospholipid syndrome, anticoagulant therapy was started after operation. She had no symptoms of thrombopenia and hemorrhage in the other organs after the start of anticoagulant therapy.

### Pathological Findings

**Figure 2:**
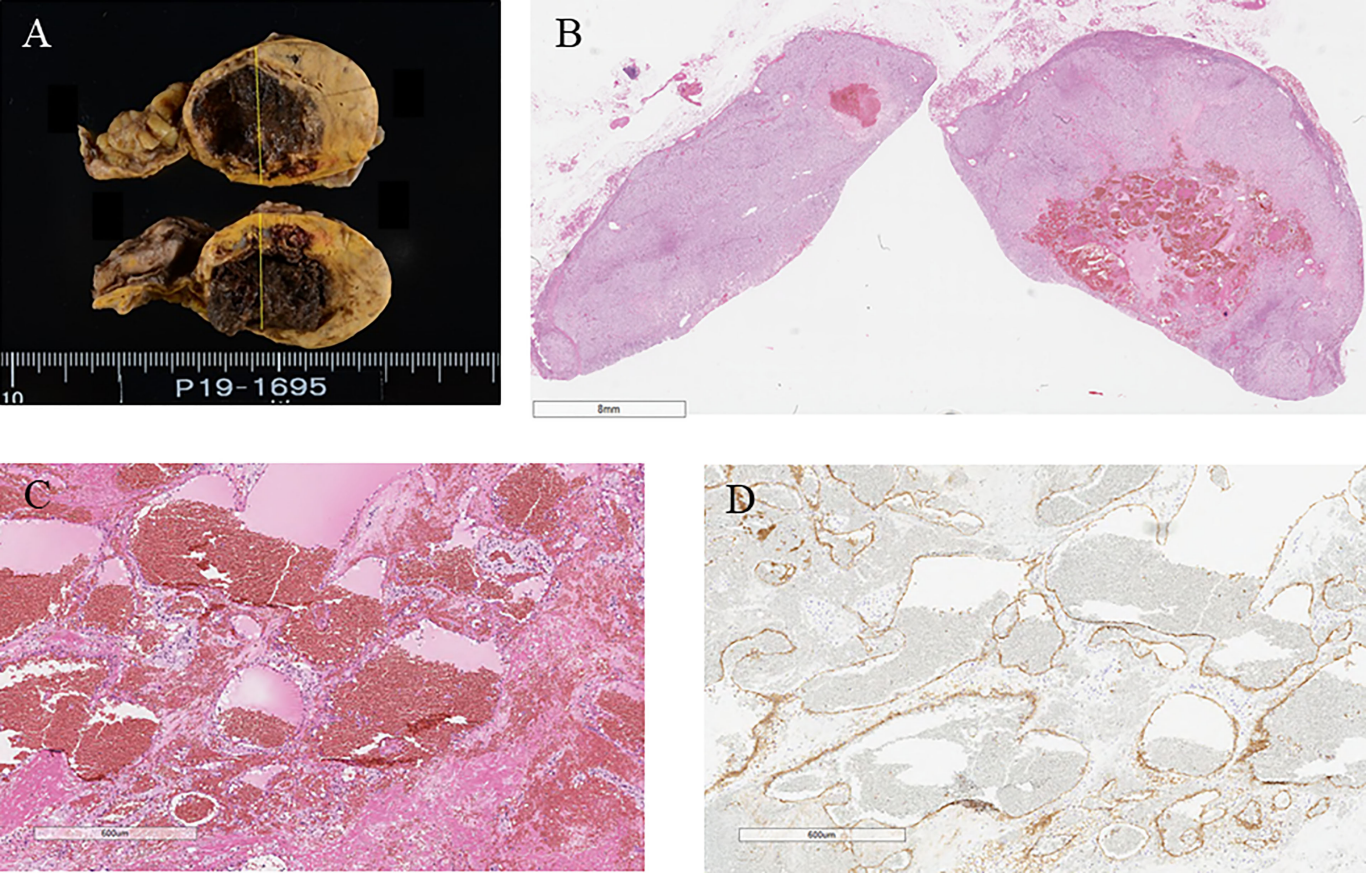
**(A)** Surgical specimen showing hemorrhage in the adrenal tumor. **(B)** Hematoxylin and eosin-stained tumor section showing hemorrhage in the tumor. **(C)** Hematoxylin and eosin-stained section on high magnification showing hemorrhage without vasculitis and ominous findings of infection (40×). **(D)** CD31 immunostaining (40×).

**Figure 3:**
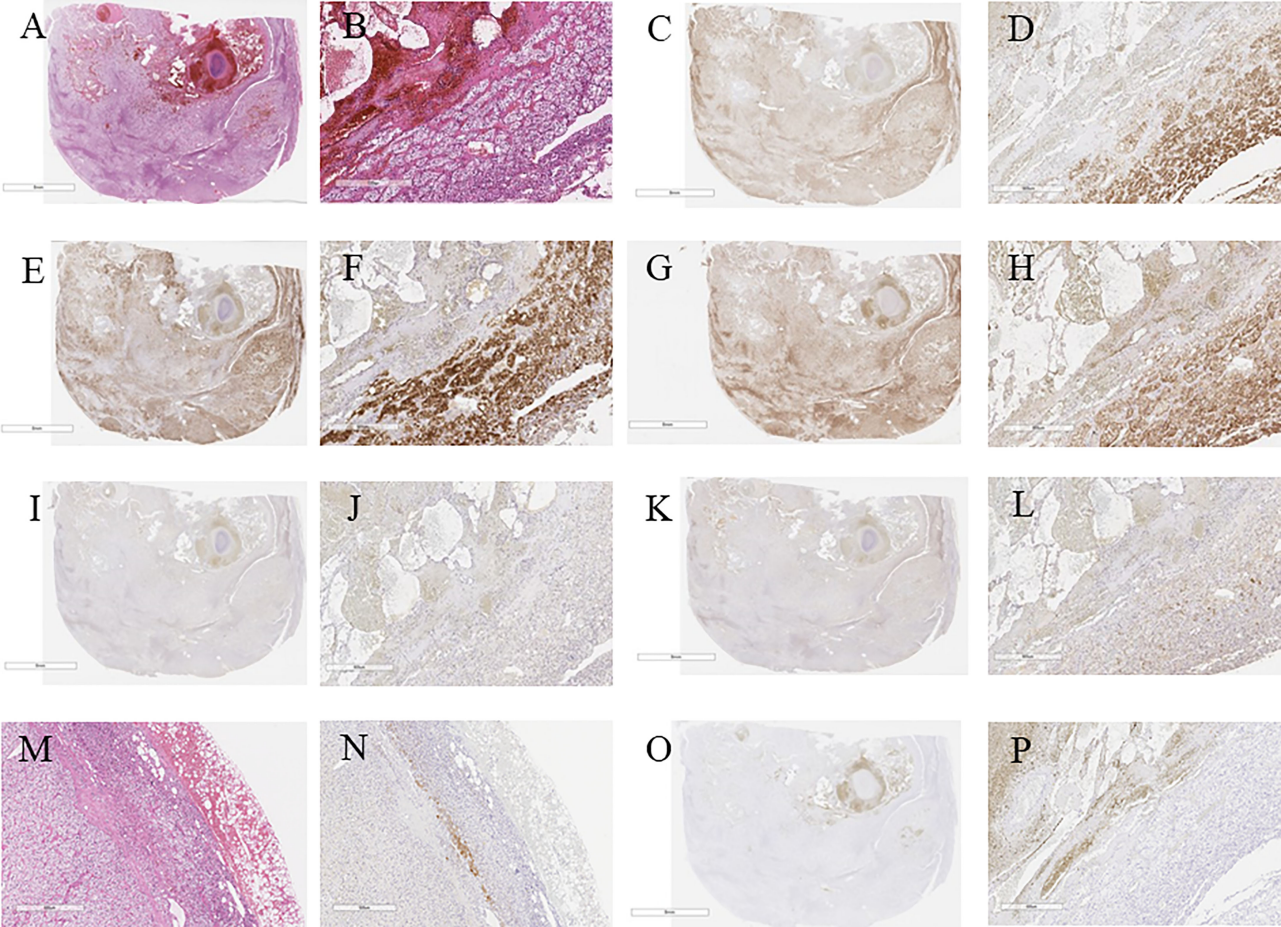
**(A)** Hematoxylin and eosin immunostaning. **(B)** Hematoxylin and eosin-stained tumor section on high magnification showing cortical adenoma (40×). **(C)** c17 immunostaining. **(D)** c17-stained tumor section on high magnification (40×). **(E)** HSD3B2 immunostaining. **(F)** HSDB2-stained tumor section on high magnification (40×). **(G)** CYP11B1 immunostaining. **(H)** CYP11B1-stained tumor section on high magnification (40×). **(I)** HSDB1 immunostaining. **(J)** HSDB1-stained tumor section on high magnification (40×). **(K)** DHEA-ST immunostaining. **(L)** DHEA-ST-stained tumor section on high magnification (40×). **(M)** Hematoxylin and eosin-stained concomitant adrenal tissue on high magnification (40×). **(N)** High magnification of DHEA-ST-stained concomitant adrenal tissue on high magnification (40×). **(O)** CYP11B2 immunostaining. **(P)** CYP11B2-stained tumor section on high magnification (40×).

Macroscopically, a well-circumscribed yellow nodule of the adrenal gland was detected in the resected specimen (**Figure 2A**). Microscopically, the nodule is composed of large polygonal tumor cells with abundant foamy clear cytoplasm and focally small compact cells composed of eosinophilic cytoplasm arranged in nests, cords, and trabeculae. Mitotic figures were not detected, and the Ki-67 labeling index was <2% in the tumor. The criteria of Weiss (1/9: clear cells in the cytoplasm were only detected) revealed that the tumor was an adrenocortical adenoma (9). In addition, fresh hemorrhage and organizing hematoma were histologically detected within the adenoma above. Fibrin thrombus was formed in the small vessels in the tumor. CD31-positive endothelial cells encroached the thrombus above and recanalization in the blood vessels inside the tumor. Any histological findings of infarction and inflammatory infiltration suggesting vasculitis or infection were not detected. In addition, hyalinized degenerative changes were also detected in the small vessel walls within the adenoma. These findings above were all consistent with the possibility of intratumoral hemorrhage and thrombosis mainly caused by antiphospholipid syndrome (**Figures 2B–D** and **3A, B**).

The tumor cells were immunohistochemically positive for steroidogenic enzymes illustrated in the figures, including DHEA-ST and 3BHSD1 (**Figures 3C**–**L**). On the other hand, adrenocortical atrophy was not detected, but DHEA-ST immunoreactivity was diminished in the concomitant adrenocortical cells, consistent with the clinical findings that this tumor had slightly elevated autonomous secretion of cortisol (**Figures 3M, N**).

In contrast, CYP11B2-positive cells were not detected in the tumor and adjacent non-neoplastic adrenal tissue. In addition, paradoxical hyperplasia of the zona glomerulosa was not detected (**Figures 3O, P**). These findings all indicated that the tumor produced cortisol. No aldosterone-secreting cells causing primary aldosteronism were identified in the tumor and non-neoplastic adrenal tissues, which was consistent with results of clinical investigations.

## Discussion and Conclusion

One of the major causes of adrenal hemorrhage is thrombosis of the adrenal vein, resulting in occlusion, swelling, and rupture of the adrenal vein (1, 2). Antiphospholipid syndrome is an autoimmune disease and clinically associated with hypercoagulability, which results in thrombophilia through auto-antibodies against the heterologous groups of phospholipids (10). These antibodies also caused arterial/venous thrombosis in various organs. Therefore, antiphospholipid syndrome can account for adrenal hemorrhage of this case through development of adrenal venous thromosis.

When diagnosing adrenal hemorrhage, imaging, such as abdominal CT, MRI, ^131^I-adosterol adrenal scintigraphy, has been generally recommended to evaluate its morphological features (11). In our case, adrenal incidentaloma was suspected by abdominal CT, abdominal MRI, and ^131^I-adosterol adrenal scintigraphy. Abdominal CT and MRI revealed the intratumoral heterogeneity inside the tumor, which eventually turned out to be due to adrenal hemorrhage. Nevertheless, the patient did not complain of any abdominal pain. As for the ^131^I-adosterol adrenal scintigraphy, an adrenal gland with hemorrhage was reported to commonly demonstrate dissipation or decreased adosterol accumulation because of the impaired cortisol secretion. However, in our case, the adosterol accumulation on the right adrenal gland was markedly detected, while it was diminished on the left adrenal gland. In addition, her cortisol level became lower after tumor extirpation. Therefore, we considered the possibility that the right adrenal tumor could harbor autonomous cortisol secretion despite the fact that preoperative laboratory data was not necessarily diagnostic of full-brown CS and SCS.

The treatment of adrenal hemorrhage is not standardized because the clinical phenotypes varied (2). Izabela et al. demonstrated that patients with a shrinking tumor should not be the candidates for surgery (12). This is because some cases of the adrenal hemorrhage spontaneously reabsorb and recover from the hematoma, which makes follow-up imaging revaluation, such as abdominal MRI or CT, clinically justified. However, when the tumor enlarges, as in our present case, adrenalectomy may be warranted due to the potential of rupture. In addition, the case in our study had concomitant antiphospholipid syndrome. Espinosa et al. reported that patients having antiphospholipid antibody treated with anticoagulation therapy had a better prognosis than those without because antiphospholipid syndrome causes noninflammatory thrombotic microangiopathy in multiple organs (13). Satta et al. demonstrated a case in which adrenal insufficiency was the first clinical symptom of antiphospholipid syndrome and represented deep vein thrombosis 7 months after the diagnose of adrenal insufficiency (14). However, anticoagulation therapy has also been reported to cause adrenal haemorrhage (6). For instance, McCroskey et al. reported patients with antiphospholipid syndrome who developed adrenal hemorrhage after anticoagulation therapy (15). Hence, appropriate anticoagulant therapy should be required for patients with antiphospholipid syndrome.

In our present case, we removed the right adrenal gland because of its enlargement and internal hemorrhage, possibly caused by the cortisol-secreting adenoma. Anticoagulation therapy was safely initiated after tumor extirpation, which improved her condition. There were no clinical evidence of thrombosis development in any of the organs for 3 years after the surgery. Therefore, acute adrenal insufficiency should be considered, particularly in bilateral adrenal hemorrhage (1, 2, 16–18). In addition, unilateral adrenal hemorrhage could also lead to adrenal insufficiency (19). In our present case, adrenal insufficiency due to adrenal hemorrhage did not occur preoperatively. However, postoperative adrenal insufficiency was expected because of the diminished adosterol accumulation in the left adrenal gland when performing preoperative ^131^I-adosterol adrenal scintigraphy. We carefully administered hydrocortisone postoperatively, and the patient did not experience any episodes of adrenal insufficiency. These findings indicated that ^131^I-adosterol adrenal scintigraphy should be performed preoperatively in similar cases to further evaluate for adrenal insufficiency due to adrenal hemorrhage and excessive cortisol secretion by the tumor.

The pathological findings also indicated that the tumor was a cortisol-secreting adenoma, although the laboratory data did not fulfill the clinical criteria of full-brown CS or SCS (7). Results of the careful pathological investigation of the resected adrenal specimens may explain this discrepancy. Few adrenocortical cells were positive for DHEA-ST, indicating that the amount of cortisol secreted from this tumor was not necessarily sufficient to suppress hypothalamo-pituitary-adrenal axis. In addition, immunoprofiles of steroidogenic enzymes in our present case were also consistent with results of previously reported studies, which could be consistent with the presence of autonomous cortisol secretion (20, 21). On the other hand, there was no evidence of aldosterone-secreting cells in the adrenal tumor and extirpated concomitant adrenal tissue, which was consistent with results of pre- and postoperative endocrinological investigations. The culprit lesion in the left adrenal gland was expected to cause primary aldosteronism, despite the absence of masses on imaging, but it awaits further investigations for clarification.

Two cases of adrenal hemorrhage that occurred in patients with cortisol-secreting adenoma have been reported in the literature (22, 23). However, there have been no previous reports of adrenal hemorrhage from thrombophilia due to antiphospholipid syndrome in cortisol-secreting adenoma. In our present case, hemorrhage in the tumor possibly caused by antiphospholipid syndrome was accurately diagnosed through both pathological and clinical investigations. Antiphospholipid syndrome was reported to represent 1–5% in the general population (24). Taking the prevalence of antiphospholipid syndrome into consideration, adrenal hemorrhage due to antiphospholipid syndrome should exist to some extent as the previous reports indicated (6, 13–15). In addition, there could be adrenal hemorrhage in patients with cortisol-secreting adenoma due to antiphospholipid syndrome like our present case. Hence, this case could provide important information as to the proper diagnosis and therapy toward similar cases in future. In addition, appropriate anticoagulant therapy for patients with antiphospholipid syndrome could possibly avoid adrenal hemorrhage, but it awaits further investigations for clarification.

In summary, we reported the first case showing adrenal hemorrhage in a cortisol-secreting adenoma due to thrombophilia secondary to antiphospholipid syndrome. The lesion was diagnosed clinically and pathologically, following the diagnosis of adrenal incidentaloma and subsequent adrenalectomy. Even though the adrenal incidentaloma is diagnosed as a non-functional tumor based on specific endocrinological criteria, periodic follow-up must be performed, particularly in the cases with slight endocrine abnormalities. Our present case also highlighted the importance of histopathological evaluation of resected adrenal glands to accurately diagnose the unusual clinical and/or radiological changes detected in the adrenal.

## Data Availability Statement

The original contributions presented in the study are included in the article. Further inquiries can be directed to the corresponding author.

## Ethics Statement

Ethical review and approval was not required for the study on human participants in accordance with the local legislation and institutional requirements. The patients/participants provided their written informed consent to participate in this study. Written informed consent was obtained from the individual(s) for the publication of any potentially identifiable images or data included in this article.

## Author Contributions

KO and IA originally drafted this manuscript and prepared the figures. KO, IA, MY, YS, KT, MK, YukY, TS, TK, YT, and KK performed the clinical investigations. YutY, SH, and HS performed the pathological investigations. YF, SM, HT, and TI performed the experiments. AL, HS, and KK reviewed the manuscript. All authors contributed to the article and approved the submitted version.

## Conflict of Interest

The authors declare that the research was conducted in the absence of any commercial or financial relationships that could be construed as a potential conflict of interest.

## Publisher’s Note

All claims expressed in this article are solely those of the authors and do not necessarily represent those of their affiliated organizations, or those of the publisher, the editors and the reviewers. Any product that may be evaluated in this article, or claim that may be made by its manufacturer, is not guaranteed or endorsed by the publisher.
